# Synergistic effect of hydration and carbonation of ladle furnace aslag on cementitious substances

**DOI:** 10.1038/s41598-022-18215-7

**Published:** 2022-08-25

**Authors:** Yuanrong Yi, Wenqing Ma, Ainiwaer Sidike, Zhongle Ma, Minghang Fang, Yue Lin, Shuqi Bai, Yinguang Chen

**Affiliations:** 1 College of Ecology and Environment, Xinjiang University, Urumqi, 830046 China; 2 Key Laboratory of Oasis Ministry of Education, Xinjiang University, Urumqi, 830046 China; 3 Key Laboratory of Smart City and Environmental Modeling Autonomous Region, Urumqi, 830046 China; 4 College of Environmental and Energy Engineering, Tongji University, Shanghai, 200092 China

**Keywords:** Environmental sciences, Materials science

## Abstract

Ladle furnace slag (LFS) can undergo hydration and carbonation reactions as cement. This article explores the effect of LFS hydration and carbonation reactions on cementitious substances at different temperatures and different LFS particle sizes, determining the effect of these varying conditions on the microstructure and formation mechanism of cementitious substances. The results show that in the early stages, C_2_S and C_3_S undergo hydration to generate C–S–H gel, which then undergoes decalcification and condensation to generate CaCO_3_ and Ca-deficient C–S–H gel; the hydration reaction and carbonation reaction promote and influence each other. The increase in temperature was found to hinder the formation of CaCO_3_ from Ca^2+^ and CO_3_^2−^, thus reducing the efficiency of hydration carbonation. The increase in particle size was not conducive to the leaching of C_2_S and C_3_S to the surface of the reaction phase, which in turn reduced the degree of decalcification and polymerization of the C–S–H gel in the carbonation phase. It was concluded that the optimum LFS hydration and carbonation reactions were achieved at 20 °C and with a LFS particle sizes < 38 μm.

## Introduction

Ladle furnace slag (LFS) is an alkaline solid waste material with a high calcium content, that is produced as a by-product of secondary refining processes in the iron and steel industry^1^. The mineral phase components of LFS mainly contain calcium metasilicate (CaSiO_3_, CS), dicalcium silicate (Ca_2_SiO_4_, C_2_S), tricalcium silicate (Ca_3_SiO_5_, C_3_S) and free calcium oxide (f-CaO)^2^. LFS exhibits poor stability and poor compactness due to its high content of f-CaO. In addition, it exerts a filler effect that can increase the density of cementitious substances, as its mineral phase composition is similar to that of Ordinary Portland cement (OPC) and it contains the same oxides that are present in OPC, while also having the advantages of a high affinity to cement-based materials and highly abundant availability^3^. The addition of LFS to cement-based materials as a mineral additive can accelerate the hydration reaction and increase the formation of hydration products such as calcium silicate hydrate (C–S–H) gel^4^. As the most important hydration product of cement slurries, C–S–H gel is an important source of mechanical properties in cementitious materials and considerably influences the carbonation of LFS and the preparation of cement^5^.

The calcium-based substances in LFS exhibit good carbonation performance with a theoretical carbonation efficiency of 30–60%^6^. The poor stability and poor compactness of LFS can be mitigated by absorbing CO_2_ to produce stable calcium carbonate (CaCO_3_). Xu^7^ used CO_2_ to immobilize heavy metals in LFS and enhance its strength. Monkman^8^ established that the extractable CaO content of carbonated LFS was reduced by about 95% compared with uncarbonated LFS, while its strength is greatly improved when formed into mortar. Therefore, carbonation can not only effectively achieve CO_2_ storage but also improve the compressive strength of the final product. In the early stage of carbonation, the cementitious substances in LFS (C_2_S and C_3_S) undergo hydration reactions to form C–S–H gel. When comparing the changes of C–S–H gel during natural carbonation or accelerated carbonation, Auroy^9^ concluded that CaCO_3_ was more conducive to forming crystalline spherical aragonite with a relatively stable structure after accelerated carbonation. Therefore, hydration and carbonation reactions are particularly important in the process of LFS resource utilization.

Huijgen^10^ analyzed the controlling factors that affect the carbonation reaction, including particle size, temperature, solid–liquid ratio, stirring speed, and aeration rate and demonstrated that temperature and particle size are the two most important factors. Ashraf^11^ compared the carbonation process of CS, C_2_S, and C_3_S at different temperatures, reporting that the reactivity of CS samples was lower than that of C_2_S and C_3_S samples. Previous studies^12,13^ have reported that reducing the average particle size of slag can shorten the leaching channels from the metal ion core to the inner surface, thus promoting the utilization of slag and interactions between the slag-water–gas system. Suh^14^ reported that heating an anhydrous calcium silicate clinker to 200 °C resulted in a gradual increase in Q_1_ structure (SiO_4_ tetrahedra connected to a SiO_4_ tetrahedra) and Q_2_ structure (SiO_4_ tetrahedra connected to two SiO_4_ tetrahedra) peak intensities, according to deconvolution of the recorded ^29^Si solid-state nuclear magnetic resonance (NMR) spectra. Suh’s results show that the hydration reaction of anhydrous calcium silicate can be accelerated by increasing the reaction temperature within a certain range. The aforementioned studies have generally assessed the effects of hydration and carbonation reactions in isolation on mineral phase composition, with only a few studies having combined both reactions to analyze the change mechanism of the cementitious substances under varying temperature and particle size conditions.

In this study, changes in the hydration reaction of C_2_S and C_3_S in LFS and the carbonation reaction of C–S–H gel when applying different temperatures and LFS particle sizes, were explored using solid-state ^29^Si NMR technology. The samples were characterized by scanning electron microscopy (SEM), thermogravimetry (TG), differential thermogravimetry (DTG), X-ray diffraction (XRD) and Brunner–Emmet–Teller (BET) analysis of nitrogen adsorption–desorption isotherms. Peak Fit software was used to analyze the changes in the cementitious substances during the hydration and carbonation stages and revealed their mechanism of microstructure formation. The present study aimed to understand the effect of different temperatures and particle sizes on cementitious substances subjected to hydration and carbonation reactions, providing theoretical guidance for the application of LFS and LFS-based cement materials.

## Materials and methods

### Experimental materials

**Figure 1:**
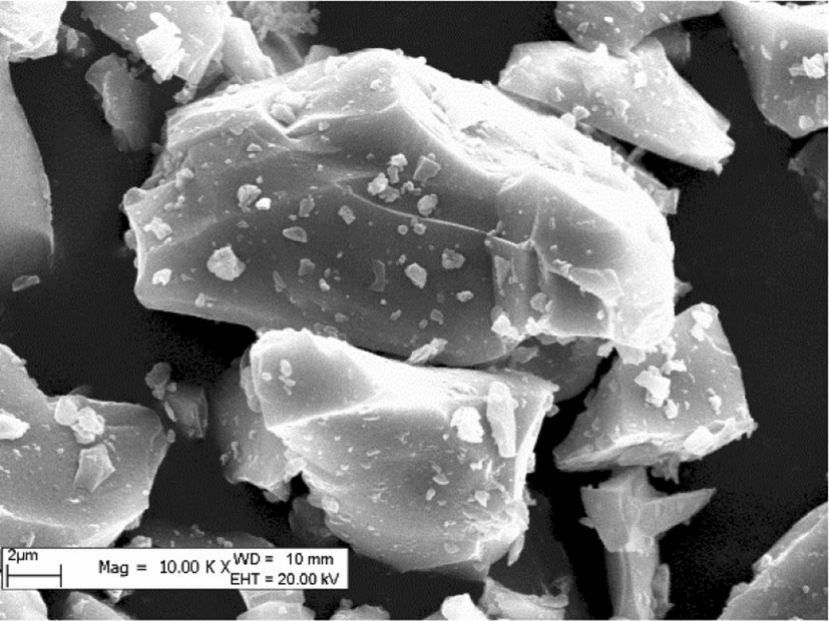
Micrograph of the original LFS material.

The LFS used in this study was taken from a steel plant in Xinjiang (China), with the chemical composition and morphology of the material shown in Table 1 and Fig. 1, respectively. XRD analysis showed that the main mineral phase components of LFS include CS, C_2_S, C_3_S and CaAl_2_Si_2_O_8_. The solid LFS material was pulverized using a jaw crusher (BB200, Retsch, Germany) and then ground with a vibrating disc grinder (RS200, Retsch, Germany). Varying particle sizes were obtained by passing the ground sample through 180 μm, 96 μm, 48 μm, and 38 μm pore size sieves before experiments.

**Table 1:** Chemical composition (by % weight) of the original LFS.

Component	CaO	Al_2_O_3_	SiO_2_	MgO	F	TiO_2_	Na_2_O	Fe_2_O_3_	MnO	Others
Content	62.32	18.64	8.45	4.26	3.65	0.81	0.083	0.746	0.20	0.83

### Experimental process

A three-phase reaction tank was employed to investigate the influence of particle size and temperature on the carbonation of LFS. Slurries of LFS fractions containing each respective particle size range, were prepared with a solid–liquid ratio of 1:5, for use in experiments at a CO_2_ ventilation rate of 500 mL/min, with continual stirring at 700 rad/min. Hydration and carbonation reactions were considered to be complete when the mass remained constant. To determine the influence of particle size, slurries with LFS particles sizes of 96–180 μm, 48–96 μm, 38–48 μm and < 38 μm were used at a reaction temperature of 20 ℃. To analyze the influence of temperature, samples with a particle size < 38 μm were reacted at 20 ℃, 40 ℃, 60 °C and 80 °C. At the end of the experiment, the solids in the slurry were separated from the liquid and dried at 105 °C for 8 h to obtain carbonation products for analysis.

### Characterization methods

X-ray diffraction analysis was performed to identify carbonation products using an X-ray powder diffractometer (D8 Advance, Bruker, Germany). For all materials, two samples were analyzed under the following experimental conditions: Cu Kα radiation (λ = 0.154 nm), scanning range of 10°–70° and a scanning rate of 2.0°/min. The mineral phase composition was determined via thermal stability analysis (TG-DTG) using a simultaneous thermogravimetric analyzer (STA 7300, Hitachi, Japan) with 10 mg of sample analyzed at a temperature range from ambient temperature to 900 °C at a heating rate of 10 °C/min in a single measurement, as variations in outcome were expected to be small^15^. A scanning electron microscope (LEO 1430VP, Carl Zeiss, Germany) was used at an accelerating voltage of 15 kV to analyze the micro-morphology of samples, which were sprayed with gold before the test to stabilize images. Analysis of the microstructure changes during C_2_S and C_3_S carbonation was carried out via ^29^Si NMR analysis (600 M spectrometer, Agilent, US), with a 4-mm ZrO2 rotor at a rotation speed of 8 kHz and TMS solution as a ^29^Si chemical shift calibration reference.

## Results and discussion

### XRD analysis of the hydration and carbonation reaction

**Figure 2:**
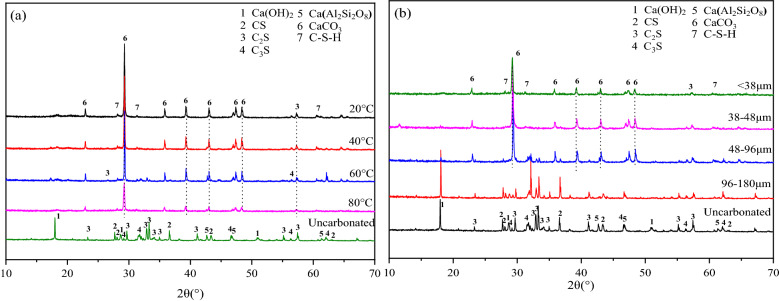
XRD patterns of original LFS and LFS samples after hydration and carbonation: (**a**) at different reaction temperatures, (**b**) at different particle sizes.

Figure 2a shows the XRD patterns of LFS samples (< 38 μm) hydrated and carbonated at different temperatures, while Fig. 2b shows the XRD patterns of LFS samples of different particle sizes after reaction at 20 ℃. These results confirm that the LFS solid waste material is composed mainly of silicate minerals, such as CS, C_2_S, and C_3_S, with additional amounts of calcium hydroxide Ca(OH)_2_ and anorthite (CaAl_2_Si_2_O_8_). After the hydration and carbonation reaction processes, the diffraction peaks of the calcium silicate phases decreased or disappeared. Simultaneously, three diffraction peaks appeared at around 2θ = 28.5°, 31.2°, and 61.2°, which were assigned to C–S–H gel^16–19^, indicating that the calcium silicate phases formed C–S–H gel during the hydration and carbonation reaction, and LFS has potential pozzolanic activity, Si and Al will also react with Ca(OH)_2_ to form C–S–H gel. As C_2_S and C_3_S are known to undergo hydration reactions^20^, C_2_S and C_3_S present on the surface of LFS particles can undergo hydration to form C–S–H gel in the early stage of the reaction. As the reaction progresses, the C–S–H gel releases Ca^2+^ to maintain the required alkaline environment^21^, resulting in decalcification of the C–S–H gel. CO_2_ gas was continuously passed through the solution to generate H_2_CO_3_ of weak acidity, which is extremely unstable and rapidly ionize in solution to generate HCO_3_^−^ and CO_3_^2−^. The free Ca^2+^ in the solution combines with CO_3_^2−^ to form stable CaCO_3_. C–S–H gel will eventually provide more Ca^2+^ than the present Ca(OH)_2_ when cement paste is exposed to high concentrations of CO_2_^22^, Finally, Ca^2+^ in CaCO_3_ mainly originates from the C–S–H gel hydration product and since the presence of moisture is a necessary condition to initiate the carbonation reaction^23,24^, hydration will occur prior to the carbonation reaction. The XRD patterns in Fig. 2 indicate the formation of large amounts of CaCO_3_ in the reaction at different temperatures and different LFS particle sizes, indicating that the main product after carbonation was CaCO_3_. As CS cannot undergo hydration^11,25,26^, the CS present on the surface of LFS particles does not form C–S–H gel in the early stage of the reaction, but instead directly combines with CO_3_^2−^ in solution to form CaCO_3_. In addition, the diffraction peaks for CaAl_2_Si_2_O_8_ did not change significantly following the hydration and carbonation reactions, indicating that the anorthite structure was relatively stable and did not participate in the reaction process, which is consistent with the results reported by Ashraf^27^. With the increase in reaction temperature, the CaCO_3_ diffraction peaks gradually decreased, which may be due to the faster movement of CO_2_ molecules and expansion of the materials volume, resulting in an increase in the distance between the molecules of CO_2_ and H_2_O, weakening the intermolecular forces and hindering the generation of CO_3_^2-^^28^. In addition, the increase in temperature also caused the solubility of CO_2_ to gradually decrease^29^, ultimately leading to a reduction in carbonation efficiency. With the decrease in particle size, the diffraction peaks of the calcium silicate phases and Ca(OH)_2_ gradually decreased, which might be explained by the simultaneous increase in surface area enhancing the contact area between particles and the solution, increasing the rate of reaction occurrence. However, a considerable amount of calcium silicates and Ca(OH)_2_ did not participate in the hydration and carbonation reactions in the largest LFS particle size, resulting in an incomplete reaction.

### TG-DTG analysis of the hydration and carbonation reaction

**Figure 3:**
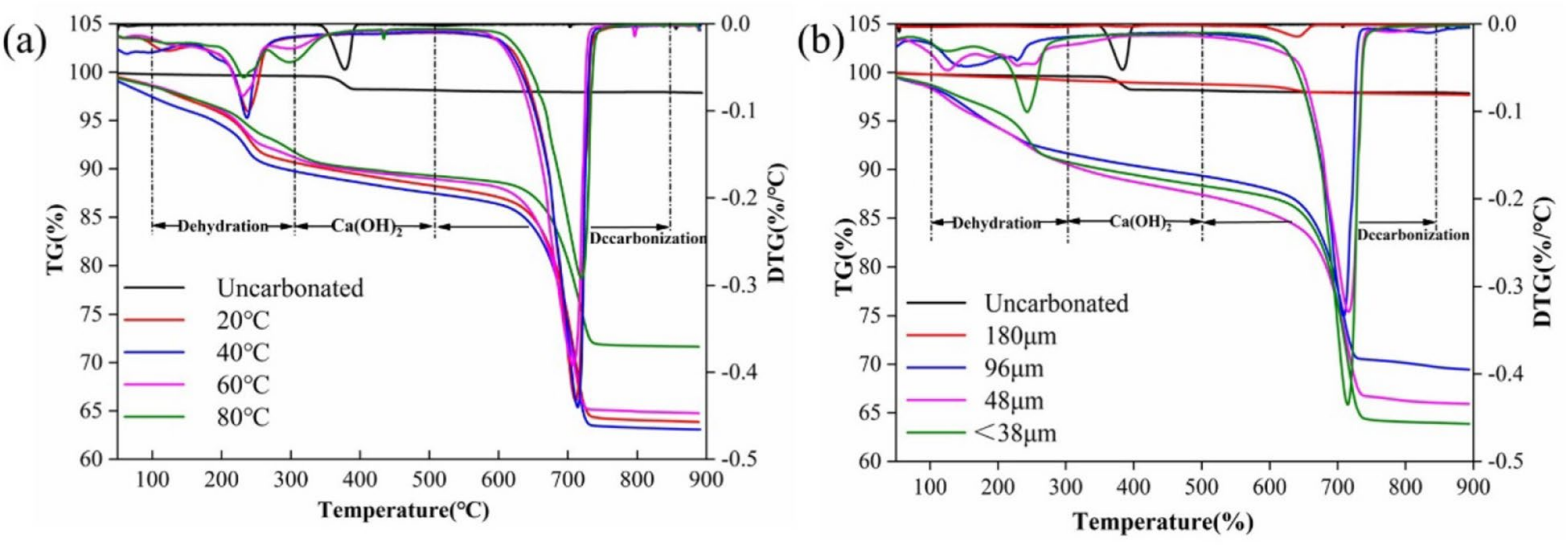
TG-DTG diagrams of the original LFS material and LFS samples after hydration and carbonation: (**a**) at different temperatures, (**b**) at different particle sizes.

Figure 3a shows the TG-DTG diagrams of LFS samples (< 38 μm) after hydration and carbonation at different temperatures, while Fig. 3b shows the TG-DTG diagrams of LFS samples of different particle sizes after hydration and carbonation reactions were performed at 20 ℃. As shown in Fig. 3, the mass losses were divided into three distinct temperature ranges: (1) 100–300 °C, which was attributed to dehydration of the C–S–H gel and the Ca-deficient C–S–H gel^30^; (2) 300–500 °C, which was attributed to the dehydration of Ca(OH)_2_^31^; (3) 500–850 °C, which was attributed to the decarbonization of CaCO_3_^32^. In terms of dry weight, the mass loss over the entire temperature range was referred to as the total mass loss. The mass of CaCO_3_ can be obtained from the CO_2_ content generated by CaCO_3_ decarbonization^33^. The weight-loss peak appearing in the range of 300–500 °C before the hydration and carbonation reactions may be caused by the formation of Ca(OH)_2_ due to the combination of calcium-based components of LFS with atmospheric water under natural conditions. Two weight-loss peaks appeared in the range of 100–300 °C and 500–850 °C after the hydration and carbonation reactions, indicating that the calcium based components of LFS underwent hydration and carbonation reactions successively, resulting in C–S–H gel, Ca-deficient C–S–H and CaCO_3_ being generated.

Table 2 lists the CaCO_3_ and total mass losses measured by TG analysis at different reaction temperatures or with different LFS particle sizes, showing that the mass of CaCO_3_ decreased with increasing temperature or LFS particle size. The content of CaCO_3_, C_2_S, and C_3_S was calculated using the reference intensity ratio (RIR) method. Results show that, as the temperature decreased, the C_2_S and C_3_S content reduced, indicating that increasing amounts of C_2_S and C_3_S participated in the hydration reaction, while the C–S–H gel continuously participated in the carbonation reaction, further promoting the hydration reaction. Therefore, a reaction temperature of 20 °C was more advantageous to the occurrence of the carbonation reaction. With the increase in particle size, the content of C_2_S and C_3_S increased, possibly because C_2_S and C_3_S in LFS require longer leaching channels to participate in the hydration reaction. Furthermore, this also negatively affected the later carbonation reaction. Therefore, larger particle sizes were not conducive to the subsequent occurrence of the carbonation reaction.

**Table 2:** Changes in the LFS mineral phase composition determined by TG and RIR at different hydration and carbonation reaction temperatures and particle sizes.

Carbonation periods	Uncarbonated	20 °C	40 °C	60 °C	80 °C	96–180 μm	48–96 μm	38–48 μm	< 38 μm
**TG (%)**									
CaCO_3_	–	24.7	25.1	24.6	18.0	1.0	20.0	21.8	24.7
Total	1.9	35.2	35.2	34.4	27.3	2.1	29.2	32.9	35.2
**RIR (%)**									
CaCO_3_	–	23.0	22.9	23.1	23.3	6.3	18.0	19.2	23.0
C_2_S, C_3_S	–	26.0	32.5	34.8	52.6	64.4	32.4	26.0	26.0

### SEM–EDS analysis of the hydration and carbonation reaction

**Figure 4:**
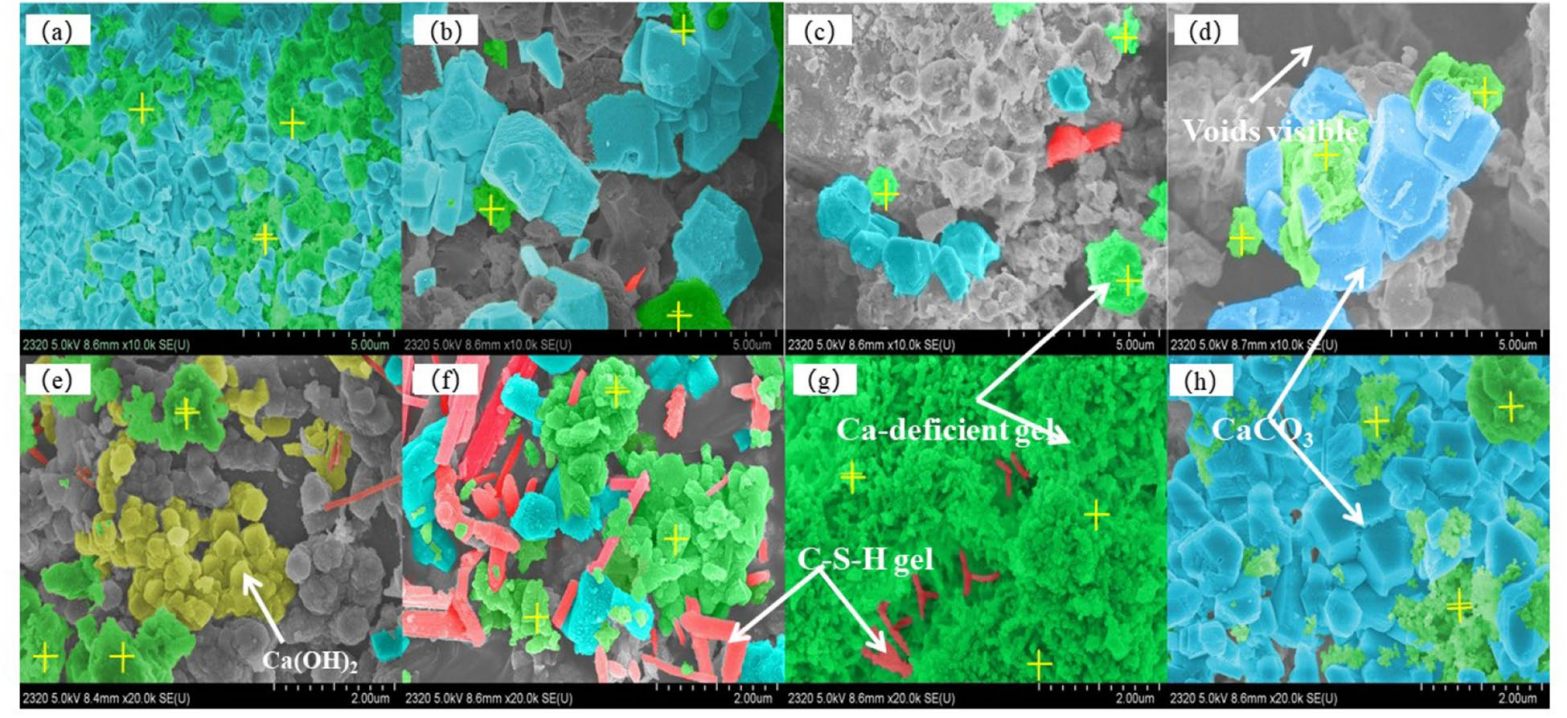
SEM images of LFS materials following hydration and carbonation under varying conditions: reaction temperatures of: (**a**) 20 ℃, (**b**) 40 ℃, (**c**) 60 ℃, (**d**) 80 ℃; LFS particle sizes of: (**e**) < 38 μm, (**f**) 38–48 μm, (**g**) 48–96 μm, (**h**) 96–180 μm.

**Figure 5:**
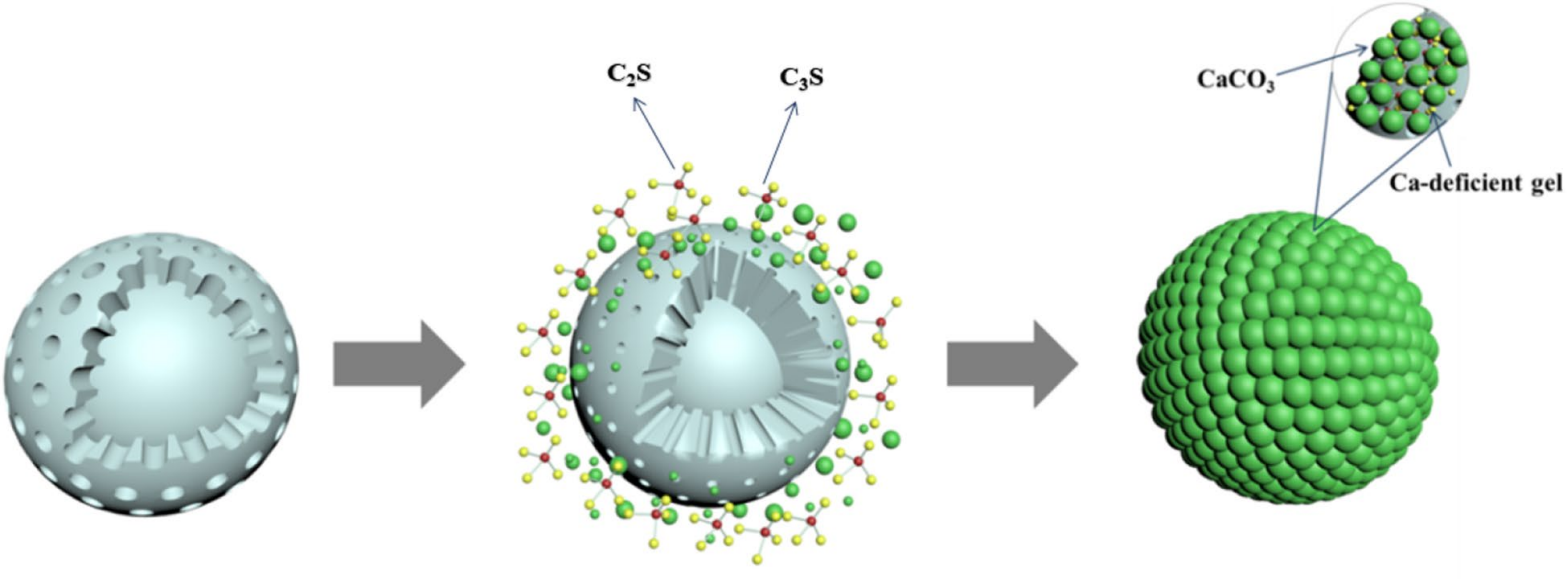
Schematic diagram of the microstructure mechanism of hydration and carbonation of LFS.

Figure 4 shows the SEM images of LFS after hydration and carbonation at different temperatures and when using samples of different particle sizes. Fig. 5 shows a schematic diagram of the microstructure mechanism of hydration and carbonation of a slag particle and Table 3 lists the average elemental content of these areas according to energy-dispersive X-ray spectroscopy (EDS). As shown in Fig. 4, after hydration and carbonation, a large amount of C–S–H gel was formed, exhibiting rough surfaces and a coagulated or fibrous form (indicated by the red area in Fig. 4), as well as CaCO_3_ crystals (such as calcite) with a cubic structure (indicated by the blue area in Fig. 4)^34,35^. As the reaction temperature increased, more voids became visible in the structure. As the LFS particle size increased, the amount of calcite gradually decreased and the LFS surface became covered by the C–S–H gel and Ca-deficient C–S–H gel (indicated by the green area in Fig. 4). When the LFS particle size was 96–180 μm, the hydration product Ca(OH)_2_ was present in the form of hexagonal flakes (indicated by the yellow area in Fig. 4). These results could be attributed to the cementitious properties of C_2_S and C_3_S, which cause the hydration reaction to form C–S–H gel and Ca(OH)_2_. During carbonation, the C–S–H gel is decalcified to form Ca-deficient C–S–H gel and CaCO_3_. Furthermore, Ca(OH)_2_ directly reacts with CO_3_^2−^ in solution to form CaCO_3_. As illustrated in Fig. 5, the carbonation products CaCO_3_ and Ca-deficient C–S–H gel adhere to the surface of the reaction phase and the entire structure becomes dense, inhibiting the diffusion of Ca^2+^ into solution, which eventually prevents further progression of the reactions. According to the results shown in Table 3, the reaction products were mainly composed of C, O, Al, Si and Ca. The content of Ca gradually increased with increases in temperature or particle size, indicating that the decalcification degree of the C–S–H gel gradually decreased and that the concentration of Ca^2+^ ions leached into the solution decreased, further reducing the formation of CaCO_3_. Therefore, increased temperature or particle size was detrimental to the hydration and carbonation reactions.

**Table 3:** Elemental content of EDS areas (%).

Samples	C	O	Al	Si	Ca
20 °C	17.3	57.8	8.3	2.3	14.3
40 °C	21.7	56.7	6.0	1.1	14.6
60 °C	30.5	47.8	3.1	0.7	18.0
80 °C	15.7	55.1	5.3	1.0	23.0
96–180 μm	7.2	54.6	11.4	2.0	25.8
48–96 μm	7.7	56.0	7.6	1.0	23.3
38–48 μm	12.7	55.7	5.8	3.1	22.8

### Microstructure of the cementitious substances in the hydration and carbonation reaction

**Figure 6:**
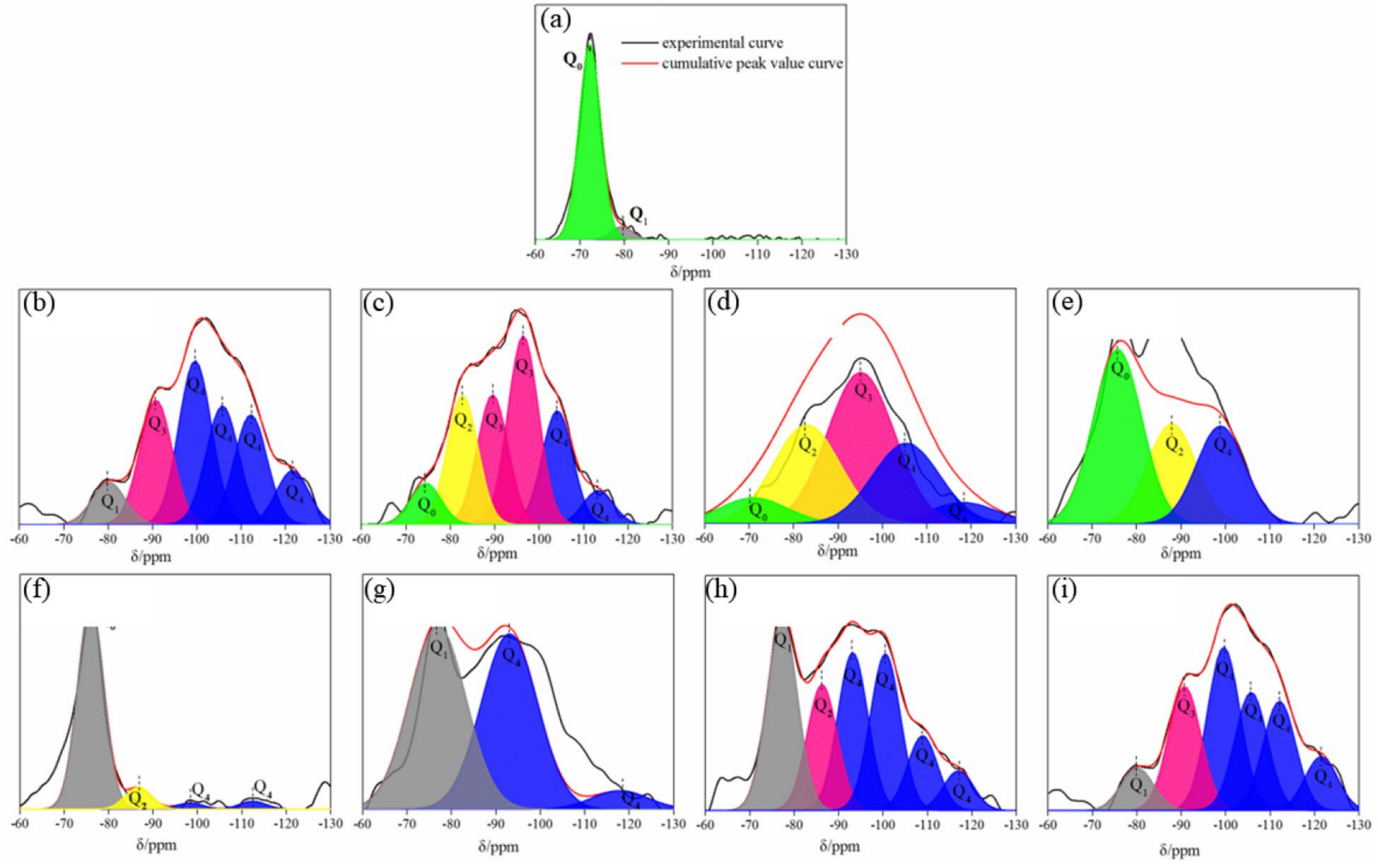
^29^Si NMR spectra of original LFS: (**a**), and LFS samples after hydration and carbonation: (**b**) at 20 ℃, (**c**) at 40 ℃, (**d**) at 60 ℃, (**e**) at 80 ℃, (**f**) at < 38 μm, (**g**) at 38–48 μm, (**h**) at 48–96 μm, (**i**) at 96–180 μm.

Table 4 shows the ^29^Si NMR chemical shift range of Q_n_ structural units in silicates^36^, while Fig. 6 shows the ^29^Si NMR spectra following hydration and carbonation of LFS at different temperatures or when using different LFS particle sizes. In NMR analysis, Q_0_ represents isolated SiO_4_ tetrahedra, Q_1_ often appears at the end group of a straight chain, Q_2_ mostly appears as a middle group of a straight chain, Q_3_ has a double-stranded polymer structure or a layered structure and Q_4_ represents SiO_4_ tetrahedra connected to four SiO_4_ tetrahedra, in a three-dimensional network structure^37^. As shown in Fig. 6a, the main peak of uncarbonated LFS was Q_0_, with a few Q_1_ peaks appearing around -79 ppm, which may be due to the formation of the C–S–H gel by C_2_S and C_3_S hydration under natural conditions. As shown in Fig. 6b–i, after the hydration and carbonation reactions, Q_2_, Q_3_, and Q_4_ peaks were present.

**Table 4:** Range of ^29^Si NMR chemical shifts of Q_n_ structural units in silicate.

Types	Chemical shift/ppm
Q_0_	− 68 to − 76
Q_1_	− 76 to − 82
Q_2_	− 82 to − 88
Q_3_	− 88 to − 98
Q_4_	− 98 to − 129

Table 5 lists the calculated deconvolution results from ^29^Si NMR analysis. No Q_3_ and Q_4_ peaks were found to be present before the hydration and carbonation reactions, indicating that it is more difficult to complete C_2_S and C_3_S hydration and carbonation reactions under natural conditions. After the hydration and carbonation reactions, the content of Q_0_ gradually decreased or even disappeared, while the Q_2_ peak appeared, indicating that at this point C_2_S and C_3_S began to participate in the reaction due to the action of the hydration reaction, resulting in the formation of a C–S–H gel with longer chain lengths. Since the Si atom radius is smaller than the Ca atom radius, the bond length of the Si–O bond is shorter than that of the Ca–O bond, resulting in the bond energy of the Si–O bond being greater than that of the Ca–O bond. Furthermore, Si exhibits weak non-metal properties, making it difficult to form ions alone in compounds. Therefore, as the reaction time increased, the Ca–O bonds in the C–S–H gel were gradually broken under the action of the carbonation reaction, while the decalcification of the C–S–H gel results in Ca^2+^ combining with CO_3_^2−^ in solution to form CaCO_3_. After decalcification, the C–S–H gel was negatively charged due to the loss of Ca^2+^. To maintain the charge balance, H^+^ in solution is adsorbed by the C–S–H gel and combines with the broken Si–O– to form –Si–OH via protonation, which subsequently undergoes a condensation reaction with the adjacent –Si–OH. This condensation increases the mean silicate chain length and forms bridges between neighboring regions, thus pulling them closer together and causing shrinkage. This results in the formation of Q_3_ and Q_4_ structures of Ca-deficient C–S–H gel with longer chain lengths and a higher degree of polymerization. CS is composed of chains of SiO_4_ tetrahedra (Q_2_), which can directly form CaCO_3_ during the carbonation process^11,38^. Equations (1), (2), and (3) were used to express the degree of decalcification (L_d_), the degree of polymerization (Pol) of the C–S–H gel, and the degree of hydration (H) of C_2_S and C_3_S, respectively^5,39^, as follows:

**Table 5:** Results of ^29^Si NMR spectra deconvolution following LFS hydration and carbonation at different reaction temperatures or particle sizes.

Samples	Unhydrated	C–S–H gel		Ca-deficient gel		L_d_	H (%)	Pol
	Q_0_ (%)	Q_1_ (%)	Q_2_ (%)	Q_3_ (%)	Q_4_ (%)			
Uncarbonated	93.8	6.2	–	–	–	0	6.2	0
20 °C	–	7	–	20.3	72.7	13	100	0.9
40 °C	6.7	–	18.1	51.4	23.8	4	93.3	0.8
60 °C	6.4	–	26.3	40.5	26.8	3	93.6	0.7
80 °C	46.8	–	26.9	–	26.3	1	53.2	0.5
96–180 μm	85.2	–	9.1	–	5.7	0.6	14.8	0.4
48–96 μm	–	48.6	–	46.6	4.8	1.1	100	0.5
38–48 μm	–	25.8	16.8	21.1	36.3	1.3	100	0.6
< 38 μm	–	7	–	20.3	72.7	13	100	0.9

1 $${L}_{d}=\frac{{Q}_{3}+{Q}_{4}}{{Q}_{1}+{Q}_{2}}$$

2 $$\mathrm{Pol}=\frac{{Q}_{3}+{Q}_{4}}{{Q}_{1}+{Q}_{2}+{Q}_{3}+{Q}_{4}}$$

3 $$\mathrm{H}=100-{Q}_{0}$$

**Figure 7:**
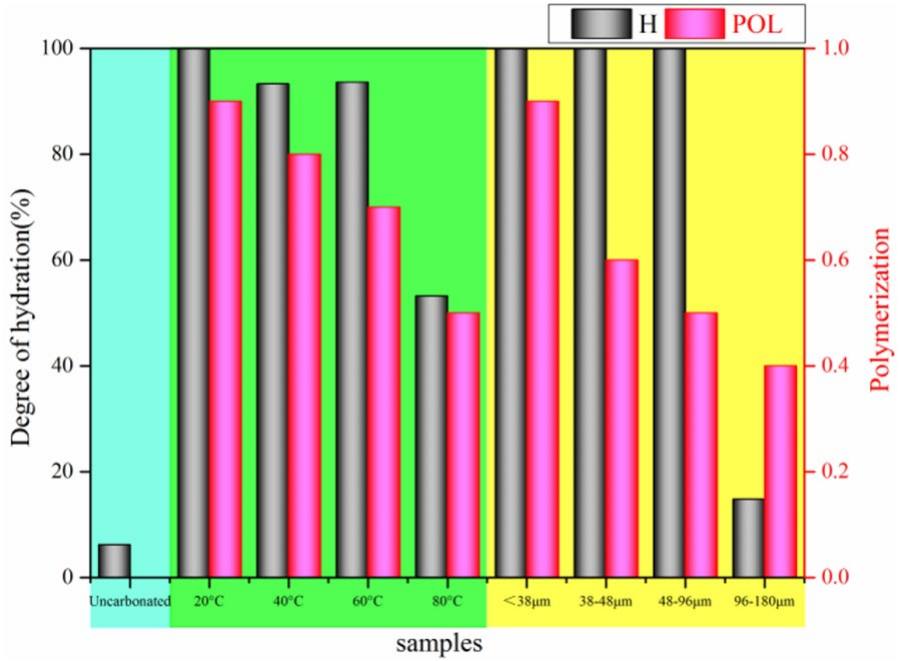
Hydration and polymerization degree of C_2_S and C_3_S at different reaction temperatures or with different LFS particle sizes.

While H reflects the degree of C_2_S and C_3_S hydration, L_d_ reflects the degree of separation of Ca^2+^ in the C–S–H gel and Pol reflects the degree of re-polymerization of the SiO_4_ tetrahedra after decalcification of the C–S–H gel. According to the results shown in Table 5, the ratio of Ca-deficient C–S–H gel to the remaining C–S–H gel did not change significantly in the temperature range of 40–80 °C and with LFS particle sizes from 38 to 180 μm, with the decalcification degree of the C–S–H gel remaining low. When the reaction conditions included a temperature of 20 °C and LFS particle sizes of < 38 μm, L_d_ was significantly increased to 13, indicating that more Ca^2+^ leached into the solution, leading to more carbonation in the rapid carbonation phase. Figure 7 shows H and Pol at different reaction temperatures and with different particle sizes, indicating that, as the reaction temperature increased, both parameters exhibited a decreasing trend. This may have occurred as the reaction products cover the reaction phase surface, preventing further contact between water and the reaction phase, which ultimately inhibits the process of hydration. Furthermore, increasing the reaction temperature reduces the solubility of CO_2_, thereby inhibiting the carbonation reaction. The observed trend in Pol was negatively correlated with the LFS particle size, which may be due to the decrease in particle size causing the –Si–OH groups in Ca-deficient C–S–H to connect and form bridges more easily with adjacent Si–OH groups, with this polymerization accelerating the carbonation reaction. Furthermore, the released Ca^2+^ combines with CO_3_^2−^ in solution to form CaCO_3_, which leads to a decrease in Ca^2+^ concentration. According to Kurdowski's theory, a low concentration of Ca will accelerate the hydration reaction of C_2_S and C_3_S and generate more C–S–H gel and Ca-deficient C–S–H gel to participate in the carbonation reaction. Therefore, the carbonation reaction promotes the hydration reaction.

## Conclusions

1. According to the obtained XRD, TG-DTG and SEM results, the hydration and carbonation reaction processes start with the hydration of C_2_S and C_3_S to form C–S–H gel. To maintain the required alkaline environment, C–S–H gel undergoes decalcification to form Ca-deficient C–S–H gel and Ca^2+^, with the Ca^2+^ and CO_3_^2−^ in solution forming structurally stable CaCO_3_. The Ca-deficient C–S–H gel undergoes condensation with adjacent –Si–OH groups to maintain charge equilibrium, resulting in a Ca-deficient C–S–H gel structure with a higher degree of polymerization of Q_3_ and Q_4_ units. Although increasing the temperature is beneficial to the conversion of Ca^2+^ in C_2_S and C_3_S, it hinders the combination of Ca^2+^ with CO_3_^2−^ and thus, inhibits the hydration carbonation efficiency. Increasing the particle size was not conducive to the hydration reaction of C_2_S and C_3_S, which in turn affects the subsequent carbonation reaction. Therefore, the hydration and carbonation reactions influence and promote each other.
2. Deconvolution of the ^29^Si NMR spectra revealed that the highest degree of hydration of C_2_S and C_3_S, the largest C–S–H gel decalcification effect and the highest degree of polymerization were achieved at a temperature of 20 °C, indicating that within the investigated temperature range the hydration carbonation reaction was optimal at this temperature. Moreover, the hydration of C_2_S and C_3_S and the polymerization of C–S–H gel decreased gradually with increasing temperature, indicating that the increase in temperature was not favorable for the hydration and carbonation reactions.
3. Deconvolution of the ^29^Si NMR spectra further indicated that the highest degree of hydration of C_2_S and C_3_S and the highest level of decalcification and polymerization of the C–S–H gel were achieved when the LFS particle size was smaller than 38 μm, indicating that the hydration and carbonation reaction was optimal at this particle size. The polymerization degree of the C–S–H gel decreased gradually with increasing particle size, showing that larger particle sizes were not conducive to the hydration and carbonation reactions.
4. In future research, the effect of cementitious substances on the properties of LFS-based cement materials will be investigated.

## Data Availability

The datasets generated during and/or analyzed during the current study are available on resonable request, by contacting the corresponding author; Yuanrong Yi(yyrhyw@163.com).
